# TqPCR: A Touchdown qPCR Assay with Significantly Improved Detection Sensitivity and Amplification Efficiency of SYBR Green qPCR

**DOI:** 10.1371/journal.pone.0132666

**Published:** 2015-07-14

**Authors:** Qian Zhang, Jing Wang, Fang Deng, Zhengjian Yan, Yinglin Xia, Zhongliang Wang, Jixing Ye, Youlin Deng, Zhonglin Zhang, Min Qiao, Ruifang Li, Sahitya K. Denduluri, Qiang Wei, Lianggong Zhao, Shun Lu, Xin Wang, Shengli Tang, Hao Liu, Hue H. Luu, Rex C. Haydon, Tong-Chuan He, Li Jiang

**Affiliations:** 1 Department of Neurology, and the Ministry of Education Key Laboratory of Child Development and Disorders, The Children’s Hospital of Chongqing Medical University, Chongqing 400046, China; 2 Molecular Oncology Laboratory, Department of Orthopaedic Surgery and Rehabilitation Medicine, The University of Chicago Medical Center, Chicago, IL 60637, United States of America; 3 Ministry of Education Key Laboratory of Diagnostic Medicine, and the Affiliated Hospitals of Chongqing Medical University, Chongqing 400016, China; 4 Department of Cell Biology, the Third Military Medical University, Chongqing 400038, China; 5 Department of Biostatistics and Computational Biology, University of Rochester, Rochester, NY 14642, United States of America; 6 School of Bioengineering, Chongqing University, Chongqing 400044, China; 7 Departments of General Surgery and Neurology, Zhongnan Hospital of Wuhan University, Wuhan 430071, China; 8 Department of Orthopaedic Surgery, the Second Affiliated Hospital of Lanzhou University, Lanzhou 730000, China; 9 Department of Orthopaedic Surgery, Shandong Provincial Hospital Affiliated to Shandong University, Jinan 250012, China; 10 Department of Surgery, West China Hospital of Sichuan University, Chengdu 610041, China; University of Cincinnati, College of Medicine, UNITED STATES

## Abstract

The advent of fluorescence-based quantitative real-time PCR (qPCR) has revolutionized the quantification of gene expression analysis in many fields, including life sciences, agriculture, forensic science, molecular diagnostics, and medicine. While SYBR Green-based qPCR is the most commonly-used platform due to its inexpensive nature and robust chemistry, quantifying the expression of genes with low abundance or RNA samples extracted from highly restricted or limited sources can be challenging because the detection sensitivity of SYBR Green-based qPCR is limited. Here, we develop a novel and effective touchdown qPCR (TqPCR) protocol by incorporating a 4-cycle touchdown stage prior to the quantification amplification stage. Using the same cDNA templates, we find that TqPCR can reduce the average Cq values for *Gapdh*, *Rps13*, and *Hprt1* reference genes by 4.45, 5.47, and 4.94 cycles, respectively, when compared with conventional qPCR; the overall average Cq value reduction for the three reference genes together is 4.95. We further find that TqPCR can improve PCR amplification efficiency and thus increase detection sensitivity. When the quantification of Wnt3A-induced target gene expression in mesenchymal stem cells is analyzed, we find that, while both conventional qPCR and TqPCR can detect the up-regulation of the relatively abundant target *Axin2*, only TqPCR can detect the up-regulation of the lowly-expressed targets *Oct4* and *Gbx2*. Finally, we demonstrate that the MRQ2 and MRQ3 primer pairs derived from mouse reference gene *Tbp* can be used to validate the RNA/cDNA integrity of qPCR samples. Taken together, our results strongly suggest that TqPCR may increase detection sensitivity and PCR amplification efficiency. Overall, TqPCR should be advantageous over conventional qPCR in expression quantification, especially when the transcripts of interest are lowly expressed, and/or the availability of total RNA is highly restricted or limited.

## Introduction

Gene expression reflects both the genetic predisposition and the physiologic condition of an individual [1–4]. By measuring gene expression, it is possible to assess an individual’s state of health and to monitor how one responds to medication, treatment, and altered living conditions. The advent of fluorescence-based quantitative real-time PCR (qPCR) has revolutionized the field of quantifying gene expression [2–4]. This technology, with its capacity to detect and measure minute amounts of nucleic acids in a wide range of samples and its combination of simplicity, speed, sensitivity, and specificity in a homogeneous assay, has been widely used in life sciences, agriculture, and medicine, as well as in molecular diagnostics including microbial quantification, gene dosage determination, identification of transgenes in genetically modified foods, risk assessment of cancer recurrence, and forensic applications [2,3].

The real-time nature of qPCR allows for collection of quantitative data as it occurs, and thus combines amplification and detection into a single step. This process is accomplished by using different fluorescent chemistries that correlate PCR product concentrations to fluorescence intensities. PCR reactions are characterized by the time point (or PCR cycle number) where target amplification is first detected because fluorescence intensity is greater than the background; this is usually referred to as cycle threshold (Cq). Consequently, the greater the quantity of target DNA in the starting material, the earlier a significant increase in fluorescent signal will appear, yielding a lower Cq value. qPCR has many benefits over other methods of quantifying gene expression. It has an accurate dynamic range of 7 to 8 log orders of magnitude and does not require post-amplification manipulation [5]. In fact, qPCR is 10,000- to 100,000-fold more sensitive than RNase protection assays [6], 1000-fold more sensitive than dot blot hybridization [7], and can even detect a single copy of a specific transcript [8]. Furthermore, qPCR analysis can detect gene expression differences as small as 23% between samples [5,9].

Although qPCR has become a standard assay in many laboratories, quantification outcomes are drastically affected by many factors, including variability of RNA templates, assay design and protocols, as well as inappropriate data normalization and inconsistent data analysis. As a result, the MIQE guidelines have been published to illustrate the essential technical steps required to generate reliable and reproducible quantification data [10]. SYBR Green-based qPCR is one of the most commonly-used platforms due to its inexpensive nature and robust chemistry [2,11]. However, the expression quantification of genes with low abundance or RNA samples extracted from highly restricted or limited sources can be challenging due to the limited detection sensitivity of SYBR Green-based qPCR analysis.

In this study, we develop a novel and effective touchdown qPCR (TqPCR) protocol by incorporating a 4-cycle touchdown stage prior to the quantification amplification stage. Using the same cDNA templates, we find that, compared with conventional qPCR, TqPCR can reduce the average Cq values for *Gapdh*, *Rps13*, and *Hprt1* reference genes by 4.45, 5.47, and 4.94 cycles, respectively, while the overall Cq value reduction for all three reference genes together is 4.95. We also find TqPCR can improve PCR efficiency and thus increase detection sensitivity. When the quantification of Wnt3A-induced target gene expression in mesenchymal stem cells is carried out, we find that, while both conventional qPCR and TqPCR are able to detect the induced expression of the relatively abundant target *Axin2*, only TqPCR can detect the up-regulation of the lowly-expressed targets *Oct4* and *Gbx2*. Furthermore, we demonstrate that the MRQ2 and MRQ3 primer pairs derived from mouse reference gene *Tbp* can be used to validate the RNA/cDNA integrity of qPCR samples. Taken together, our results indicate that TqPCR can increase detection sensitivity and PCR amplification efficiency. Thus, TqPCR should be advantageous over conventional qPCR in expression quantification when the transcripts of interest are lowly expressed, and/or the availability of total RNA is highly restricted or limited.

## Materials and Methods

### Cell culture and chemicals

Human HEK-293 line and mouse lines C2C12 and C3H10T_1/2_ were purchased from ATCC (Manassas, VA) and maintained in complete Dulbecco’s Modified Eagle’s Medium (DMEM) containing 10% fetal bovine serum (FBS, Invitrogen, Carlsbad, CA), 100 units of penicillin and 100μg of streptomycin at 37°C in 5% CO_2_ [12–17]. The immortalized mouse cell lines iMEFs, iHPs, iMACs, iCPs, and iSCAPs were previously characterized and reported [13,18–24]. The recently engineered 293pTP line was used for adenovirus amplification [25]. Both 293pTP and mouse cell lines were maintained in complete DMEM. Unless indicated otherwise, all chemicals were purchased from Sigma-Aldrich (St. Louis, MO) or Fisher Scientific (Pittsburgh, PA).

### RNA isolation and reverse transcription

Exponentially growing mouse cells (e.g., C2C12, C3H10T_1/2_, iMEFs, iHPs, iMACs, iCPs, and iSCAPs) were used for total RNA isolation with the TRIZOL Reagents RNA isolation protocol (Invitrogen). The isolated RNA was subjected to reverse transcription with hexamer and M-MuLV reverse transcriptase (New England Biolabs, Ipswich, MA). The resultant cDNA products were kept at -80°C and used as PCR templates.

To assess the quality of reverse transcription reactions on the RNA samples isolated from tissue samples, we also prepared RNA samples from dissected young adult mouse (n = 6, 4 week-old, male, CD1 strain) intervertebral disc tissues. The use and care of animals were approved by the Institutional Animal Care and Use Committee at The University of Chicago (Protocol# 71108). Briefly, the animals were euthanized and lumbar spine intervertebral disc tissues were dissected out, minced into small pieces, and lysed with TRIZOL Reagents. Total RNA was purified according to the manufacturer’s instructions, and subsequently subjected to reverse transcription as described above. The resultant cDNA products were kept at -80°C and used as PCR templates.

### Conventional Quantitative real-time PCR (qPCR)

All qPCR reactions were designed and carried out according to the MIQE guidelines [10]. The PCR primers used in this study were designed by using the web-based Primer3Plus program [26] and listed in **S1 and S2 Tables**. The PCR amplicon sizes range from 80-bp to 250-bp. The qPCR analysis was carried out by using the Opticon II DNA Engine (Bio-Rad, CA) and CFX-Connect (Bio-Rad) as described [16,18,23,25,27–34]. The qPCR reactions were done in triplicate. Briefly, the 2x SsoFast qPCR Supermix with EvaGreen or iTaq Universal SYBR Green Supermix (Bio-Rad) was used and qPCR reactions were carried out as follows: 95°C × 3’ for one cycle; 95°C × 20”, 55°C × 10”, 70°C× 1”, followed by plate read, for 40 cycles. Serial dilutions of cDNA samples were performed to determine amplification efficiency for each primer pairs. No template controls (NTCs) were used as negative controls.

### Touchdown qPCR (TqPCR)

TqPCR was carried out using the same thermocycler units, Opticon II DNA Engine and CFX-Connect, and the 2x SsoFast qPCR Supermix with EvaGreen or iTaq Universal SYBR Green Supermix. TqPCR reactions were carried out in triplicate using the following conditions: 95°C × 3’ for one cycle; 95°C × 20”, 66°C × 10”, for 4 cycles by decreasing 3°C per cycle; 95°C × 20”, 55°C × 10”, 70°C× 1”, followed by plate read, for 40 cycles. Serial dilutions of cDNA samples were performed to determine amplification efficiency for each primer pairs. No template controls (NTCs) were used as negative controls.

### Generation and amplification of the recombinant adenoviruses expressing Wnt3A and GFP

Recombinant adenovirus expressing Wnt3A was generated using the AdEasy technology as described [35–39]. The coding region of mouse Wnt3A was PCR amplified and cloned into an adenoviral shuttle vector, and subsequently used to generate and amplify recombinant adenoviruses in HEK-293 or 293pTP cells [25]. The resulting adenovirus was designated as AdWnt3A, which also expresses GFP [40–43]. Analogous adenovirus expressing only GFP (AdGFP) was used as a control [31,44–46]. For all adenoviral infections, polybrene (4–8μg/ml) was added to enhance infection efficiency as previously reported [15].

### Statistical analysis

The quantitative assays were performed in triplicate and/or repeated three times. Data are expressed as mean ± SD. All statistical tests were two-sided. P-values of less than 0.05 were considered to be statistically significant. For RQ data analysis, the correlation analyses between BRQ (Bio-Rad’s propitiatory mouse RQ1 and RQ2 primers) and all six MRQ (customized mouse RQ primers) groups were conducted respectively. The correlations between BRQ and all six MRQ groups were also visualized via scatter plots. Statistical analyses were performed using SAS version 9.3 (SAS Institute, Inc., Cary, NC).

## Results

### Incorporation of a touchdown stage expands qPCR dynamic range

**Fig 1:**
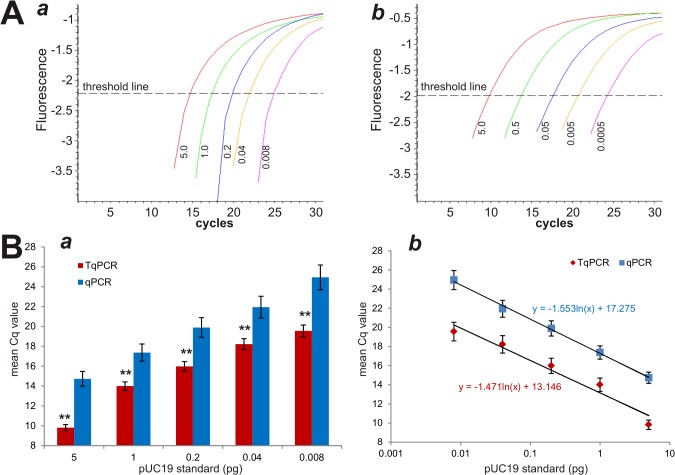
A comparison between conventional qPCR and touchdown qPCR (TqPCR) in quantitatively amplifying a target sequence in pUC19. **(A)** TqPCR expands the detection limit of DNA levels. PCR primers specific for pUC19 plasmid were used to amplify serially diluted pUC19 DNA, and the themocycling programs were carried out using a DNAEngine OPTICON II unit (Bio-Rad). PCR amplification curves (e.g., SYBR Green fluorescence history vs. cycle numbers) were plotted for conventional qPCR **(*a*)** and TqPCR**(*b*)**. **(B)** Cq value comparison between conventional qPCR and TqPCR reactions. Mean Cq values were obtained for both qPCR and TqPCR using the same dilution series of pUC19 DNA **(*a*)**. The standard curves were established with scatter plot **(*b*)**. Triplicate was performed for each assay condition. “**”, indicating the Cq difference between qPCR and TqPCR groups is significant with p<0.001.

Although SYBR-Green-based qPCR is one of the most commonly used techniques to determine gene expression levels in cells and/or tissues, the expression of genes with low transcript abundance can be challenging due to the limited sensitivity of SYBR Green detection. We sought to test if including several touchdown steps prior to quantitative amplification cycles would increase detection sensitivity. We performed a comparison study using serially-diluted pUC19 plasmid DNA samples on an older thermocycler unit DNAEngine OPTICON II. Four touchdown steps at 66°C, 63°C, 60°C and 57°C were manually added to the PCR cycling program. We found that the fluorescence values of the conventional qPCR program yielded a significantly narrower detection range than that of TqPCR, 5.0~0.008pg and 5.0~0.0005 for qPCR and TqPCR, respectively (**Fig 1A, panels *a vs*. *b***). Moreover, these data also indicate that TqPCR increases the detection sensitivity by 16-fold.

We further analyzed the Cq values of PCR reactions that were performed on the same set of templates, finding that a significantly lower Cq value was consistently observed in the TqPCR group than in the conventional qPCR group at each template concentration (p<0.001) (**Fig 1B, panel *a***). When the standard curves were analyzed on a scatter plot, we found that the calibration curves for both protocols were nearly in parallel with similar slopes (**Fig 1B, panel *b***), indicating the addition of the four touchdown steps seems to shift the standard curve downwards. These results strongly suggest that TqPCR may increase detection sensitivity.

### TqPCR protocol significantly increases the PCR amplification efficiency and the detection sensitivity, especially for low abundance transcripts

**Fig 2:**
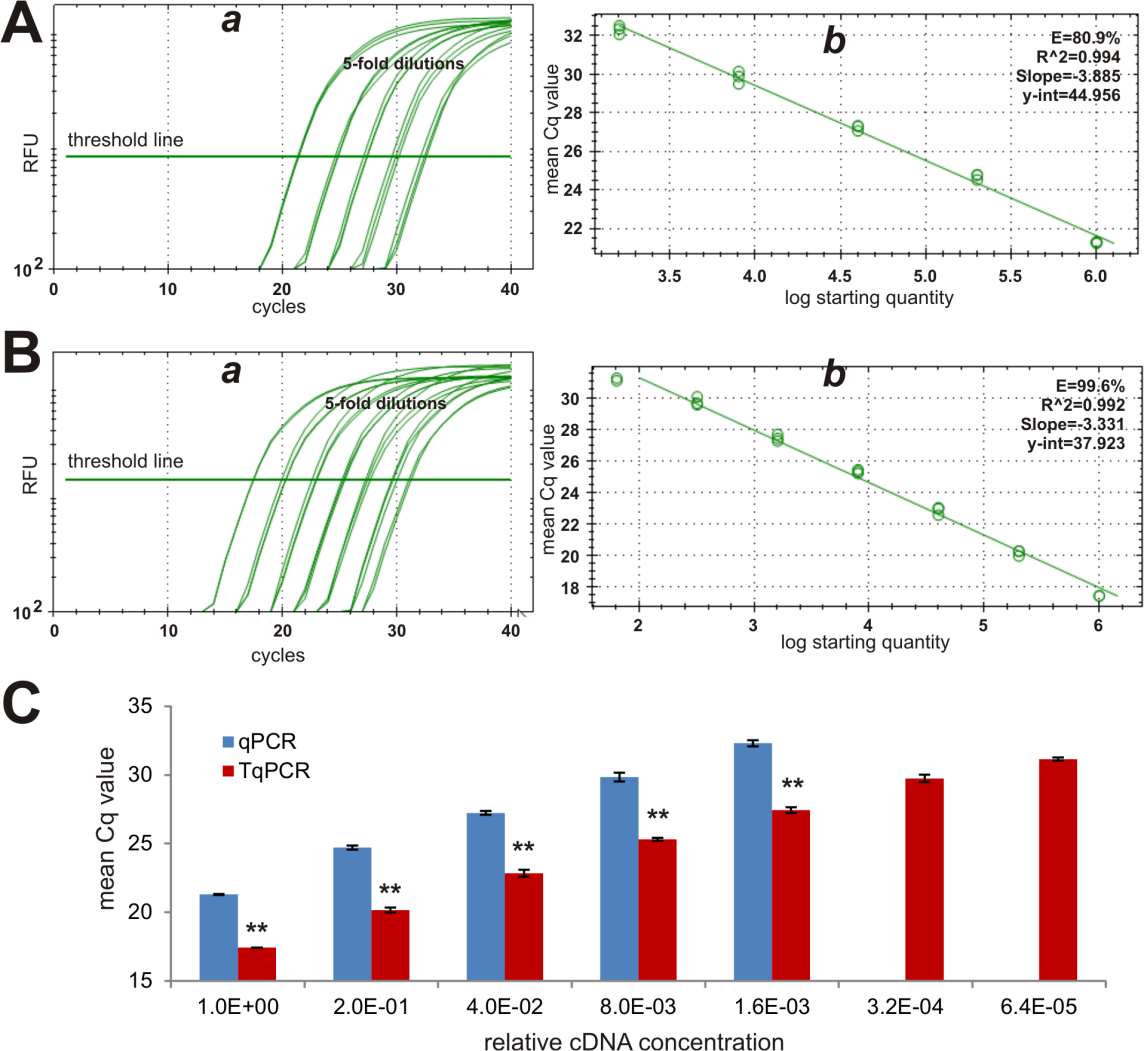
Comparative analysis of highly abundant reference gene *Gapdh* expression detected by conventional qPCR and TqPCR. The cDNA sample was prepared from the exponentially growing iMEF cells and was 5-fold serially diluted for PCR amplification. Primers specific for mouse *Gapdh* were used to detect *Gapdh* expression with the conventional qPCR **(A)** and TqPCR **(B)** protocols using CFX-Connect qPCR unit (Bio-Rad). The qPCR SYBR Green fluorescence history vs. cycle numbers **(*a*)** and the qPCR efficiencies of *Gapdh* **(*b*)** were analyzed and presented. The mean Cq value comparison of the two procedures was further graphed **(C)**. Note that the qPCR protocol did not yield reliable results for the two most diluted cDNA samples. “**”, indicating the Cq difference between qPCR and TqPCR groups is significant with p<0.001.

The amplification efficiency is critical to the accurate interpretation of qPCR data. The ideal efficiency should be 100%, which means that during the logarithmic phase of the reaction, the PCR product of interest is doubled with each cycle. For example, a perfect PCR efficiency will show a change of 3.3 cycles between 10-fold serial dilutions of a template. However, the amplification efficiency can be significantly affected by the quality of the templates, primers, and fluorescence chemistry, leading to the efficiency below or over 100%. Using a relatively new and commonly-used qPCR unit CFX-Connect, we conducted a comprehensive comparison study on the capabilities of the conventional qPCR and TqPCR protocols of detecting expression of genes with significantly different abundances. We chose three reference genes, *Gapdh*, *Rps13*, and *Hprt1* with high, medium, and low abundance, respectively [47–50]. As one of the most commonly-used reference genes, *Gapdh* should be highly expressed in most cells. When cDNA samples were prepared in 5-fold serial dilutions, Gapdh expression was readily detected by both conventional qPCR and TqPCR (**Fig 2A & 2B**). However, the relative fluorescence signal was shown to appear earlier in TqPCR than in the conventional group (**Fig 2A-*a* vs. 2B-*a***). When PCR efficiencies were analyzed and plotted, we found both protocols yielded comparable, high correlation coefficients (**Fig 2A-*b* & 2B-*b***). However, TqPCR protocol significantly increased the PCR amplification efficiency (99.6%) compared with conventional qPCR (80.9%) (**Fig 2A-*b vs*. 2B-*b***). Plotting mean Cq value clearly demonstrated that TqPCR protocol consistently yielded significantly lower Cq values than the regular qPCR protocol (p<0.001) (**Fig 2C**). The average Cq value difference between qPCR and TqPCR was 4.45. Furthermore, it’s noteworthy that the conventional qPCR failed to yield reliable amplifications for the two highest dilutions under the same conditions as TqPCR (**Fig 2A and 2C**). These results indicate that TqPCR protocol can increase amplification efficiency and hence detection sensitivity, even for abundant genes such as *Gapdh*.

**Fig 3:**
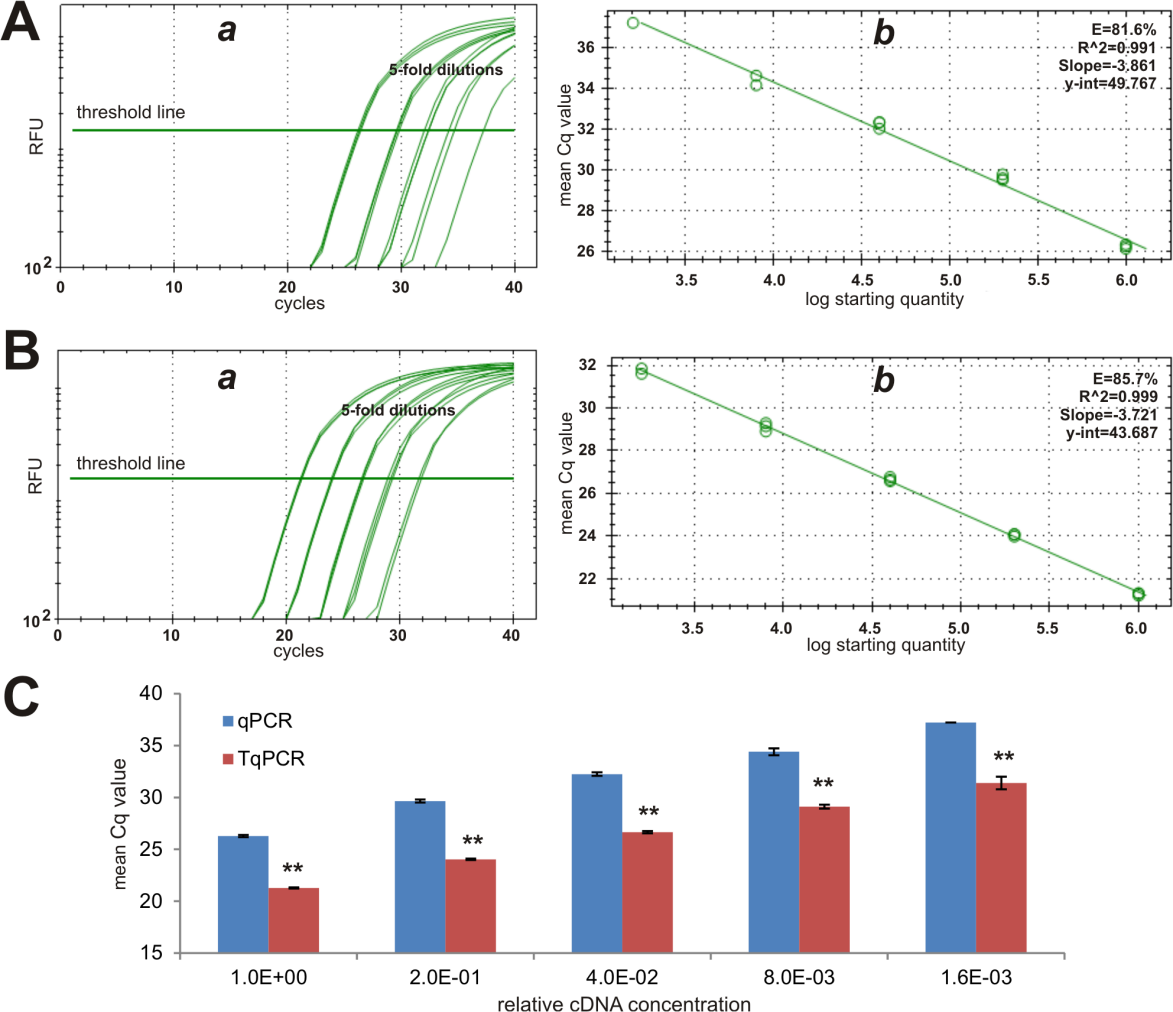
Comparative analysis of moderately abundant reference gene *Rps13* expression detected by conventional qPCR and TqPCR. The cDNA sample was prepared from the exponentially growing iMEF cells and was 5-fold serially diluted for PCR amplification, as described in Fig 2. Primers specific for mouse *Rps13* were used to detect its expression with the conventional qPCR **(A)** and TqPCR **(B)** protocols using CFX-Connect qPCR unit (Bio-Rad). The qPCR SYBR Green fluorescence history vs. cycle numbers **(*a*)** and the qPCR efficiencies of *Rps13* **(*b*)** were analyzed and presented. The mean Cq value comparison of the two procedures was further graphed **(C)**. “**”, indicating the Cq difference between qPCR and TqPCR groups is significant with p<0.001.

We next analyzed the expression of the medium abundance reference gene *Rps13*. Similar to what was observed with *Gapdh* expression, TqPCR protocol shifted the relative fluorescence signal to the left with an earlier appearance than in the qPCR group (**Fig 3A *vs*. 3B**). Furthermore, it seems that TqPCR protocol can also increase the amplification efficiency (85.7% *vs*. 81.6%) (**Fig 3A-*b vs*. 3B-*b***). The mean Cq values were consistently lower in TqPCR than in qPCR (p<0.001) (**Fig 3C**). In fact, the average Cq value difference between qPCR and TqPCR was 5.47. These results further confirm that TqPCR can improve PCR efficiency and increase detection sensitivity.

**Fig 4:**
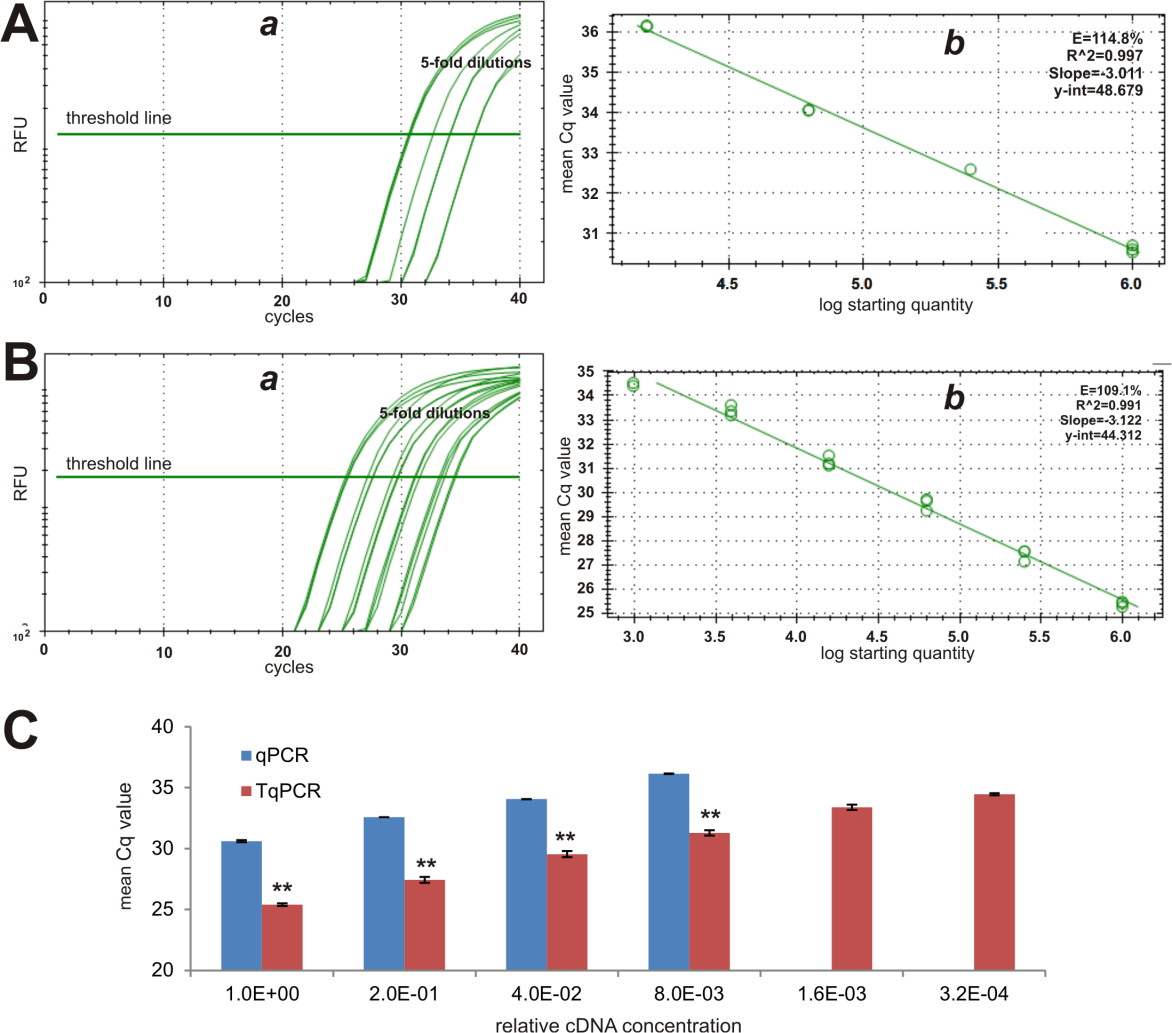
Comparative analysis of less abundant reference gene *Hprt1* expression detected by conventional qPCR and TqPCR. The cDNA sample was prepared from the exponentially growing iMEF cells and was 5-fold serially diluted for PCR amplification, as described in Fig 2. Primers specific for mouse *Hprt1* were used to detect its expression with the conventional qPCR **(A)** and TqPCR **(B)** protocols using CFX-Connect qPCR unit (Bio-Rad). The qPCR SYBR Green fluorescence history vs. cycle numbers **(*a*)** and the qPCR efficiencies of *Hprt1* **(*b*)** were analyzed and presented. The mean Cq value comparison of the two procedures was further graphed **(C)**. Note that the qPCR protocol did not yield reliable results for the two most diluted cDNA samples. “**”, indicating the Cq difference between qPCR and TqPCR groups is significant with p<0.001.

Since *Hprt1* is commonly accepted to have low abundance, it should be an ideal reference gene to test qPCR detection sensitivity. Consistent with its low expression level, the mean Cq value for the template with lowest dilution was 30.62 cycles in the qPCR group, while the Cq value was 25.40 for the same template in the TqPCR group (**Fig 4A-*a vs*. 4B-*a***). Furthermore, the TqPCR protocol improved the PCR amplification efficiency to 109.1% from 114.8% in regular qPCR (**Fig 4A-*b vs*. 4B-*b***). According to the MIQE guidelines, the ideal PCR efficiency is 100%, while the acceptable range is from 80% to 120% [10]. The mean Cq values were significantly lower in the TqPCR group than in the regular qPCR group (p<0.001) (**Fig 4C**). The average Cq value difference between qPCR and TqPCR was 4.94. It’s important to note that, unlike TqPCR, conventional qPCR failed to yield reliable Cq values for the the two highest dilutions of templates (**Fig 4A & 4C**), indicating that TqPCR has superior detection sensitivity. Taken together, the above results strongly suggest that the optimized TqPCR protocol may expand the dynamic range and detection sensitivity by improving amplification efficiencies.

### Wnt3A-induced expression of downstream target genes in mesenchymal stem cells (MSCs) is more efficiently detected by TqPCR

**Fig 5:**
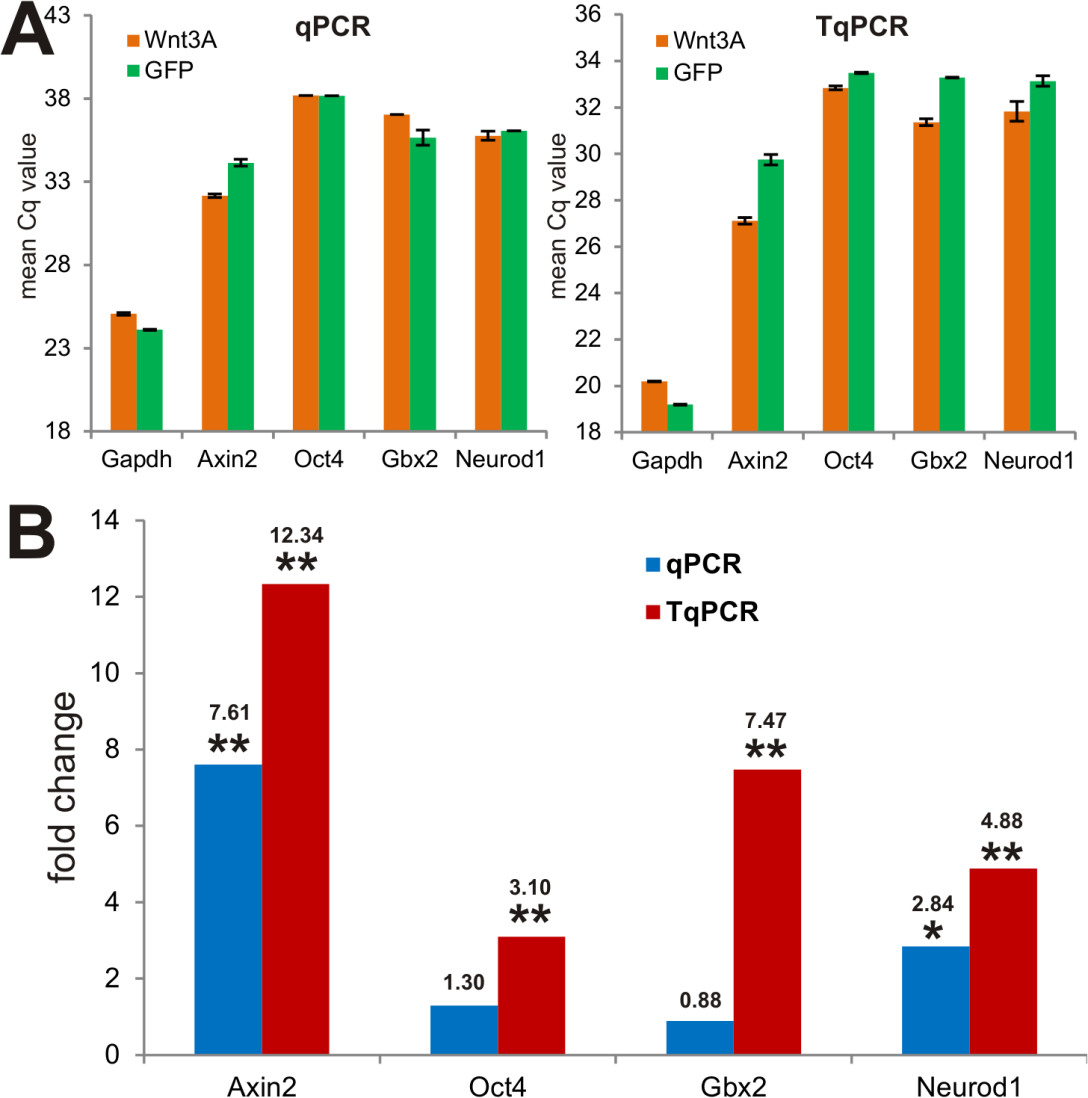
Wnt3A-induced expression of downstream target genes in mesenchymal stem cells is more efficiently detected by TqPCR. Subconfluent iMEF cells were infected with Ad-Wnt3A or Ad-GFP. Total RNA was collected at 36h after infection for reverse transcription. The resultant cDNA samples were used as templates for qPCR and TqPCR analysis using primers for *Gapdh* reference gene and several known canonical Wnt target genes, such as *Axin2*, *Oct4*, *Gbx2*, and *Neurod1*. The mean Cq values **(A)** and the fold changes in gene expression **(B)** were analyzed and graphed. The qPCR reactions were done in triplicate. The numeric values indicate the fold changes over the control group. “*”, p<0.05; “**”, p<0.001.

We next sought to determine Wnt3A-induced target gene expression in MSC cells using TqPCR vs. regular qPCR protocols. Along with many other signaling pathways, such as BMPs and IGFs [51,52], Wnt signaling plays an important role in regulating the proliferation and differentiation of MSCs [53,54]. We used a recombinant adenovirus to transduce immortalized mouse embryonic fibroblasts (iMEFs) and express Wnt3A, which should activate the canonical β-catenin/Tcf4 pathway and regulate a set of well-characterized downstream target genes, such as *Axin2* [55–57], *Oct4* [58], *Gbx2* [59] and *NeuroD1* [60]. We transduced the iMEFs with AdWnt3A or AdGFP for 36h and isolated total RNA for reverse transcription. The cDNA samples were used in qPCR and TqPCR analyses for the expression of the four target genes above and the Gapdh reference gene. We found that Wnt3A expression can upregulate these target genes to various extents (i.e., as indicated by the lower Cq values), while the *Gapdh* expression was independent of Wnt3A expression (**Fig 5A**). Specifically, while both qPCR and TqPCR analyses showed that Wnt3A up-regulated *Axin2* expression (p<0.001), only TqPCR analysis revealed the up-regulation of *Oct4* and *Gbx2* expression by Wnt3A (both p<0.001) (**Fig 5B**). In the case of *Neurod1* expression, while both qPCR and TqPCR detected the Wnt3A-dependent up-regulation of *Neurod1* expression, TqPCR analysis showed more significant up-regulation (4.88-fold, p<0.001) than conventional qPCR analysis (2.84-fold, p<0.05) (**Fig 5B**). These results further demonstrate that the TqPCR protocol is more sensitive and efficient in detecting Wnt3A-induced target gene expression, especially when the target gene abundance is low (e.g., Cq > 30).

### Characterization of RQ primers for assessing the RNA and cDNA quality for TqPCR

**Fig 6:**
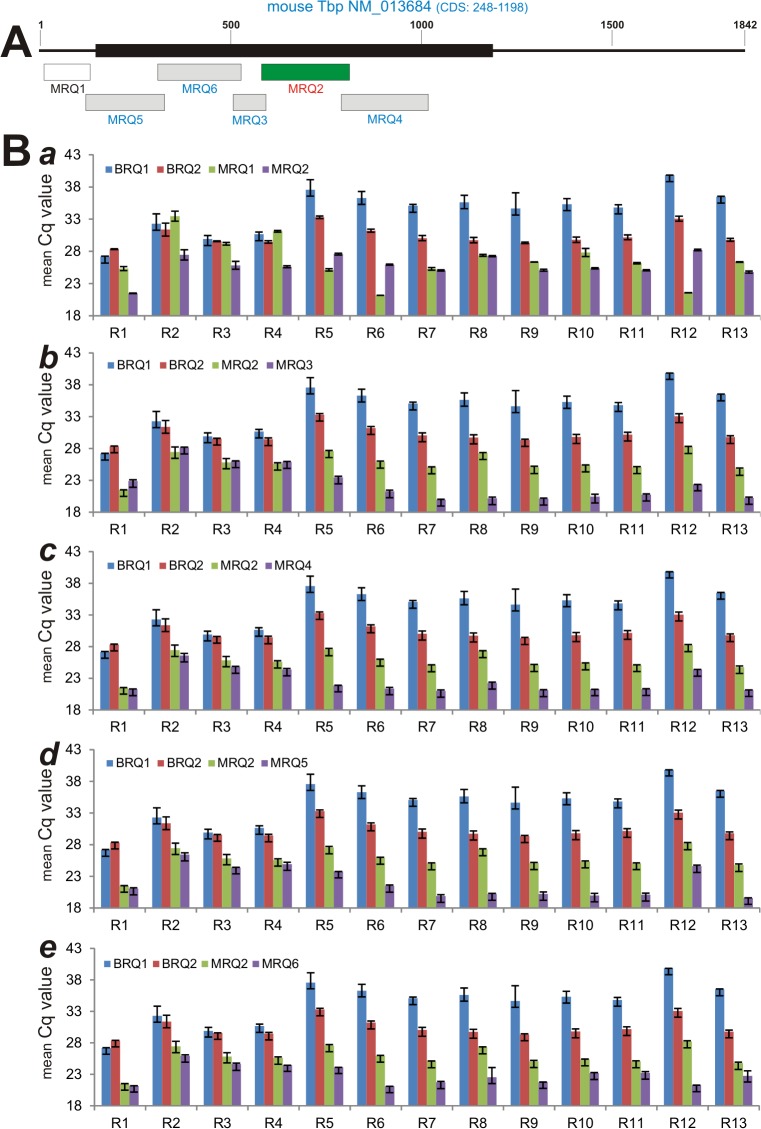
Evaluation of customized TqPCR primers that can be used to assess the RT-reverse transcription quality (RQ) of qPCR templates from mouse RNA samples. **(A)** Schematic representation of the sizes and locations of the PCR primers (also see S2 Table). **(B)** Comparison and correlation analyses of mean Cq value differences between Bio-Rad’s RQ1 and RQ2 primers (BRQ1 and BRQ2) and customized RQ primers (e.g., MRQ1 through MRQ6). The mean Cq values were obtained by performing TqPCR assay. Possible correlations between BRQ1/2 and MRQ primers were further analyzed statistically.

While the MIQE guidelines provide a comprehensive checklist for qPCR analysis [10], we found that one of the most important parameters for a successful and reproducible qPCR analysis is the quality of cDNA samples, which may be affected by RNA integrity and/or variations introduced during reverse transcription reactions. Bio-Rad offers pre-validated RQ1 and RQ2 primers (BRQs) to assess the RNA quality of a given cDNA sample, based on the principle that the Cq value differences between RQ1 and RQ2 should be identical or as minimal as possible if the RNA integrity and cDNA quality are good. In order to allow investigators to design homemade RQ primers, we sought to carry out TqPCR assays to test a panel of six home-designed RQ primers for mouse cDNA samples (MRQs) by choosing the mouse reference gene *Tbp* as the target template (**Fig 6A**).

To test how accurately the MRQ primers may assess RNA and cDNA quality when compared with the pre-validated Bio-Rad RQ primers, we prepared a panel of 13 mouse cDNA samples, seven of which (R1 through R7) were prepared from the mouse cell lines iMEFs, C2C12, C3H10T1/2, iHPs, iMACs, iCPs, and iSCAPs, respectively, and six of which were prepared from the dissected mouse lumbar intervertebral disc tissues (R8 through R13). When these MRQ primers were run side-by-side with the BRQ primers on the 13 cDNA samples using the TqPCR protocol, we found the trends of Cq changes of several MRQ primers closely correlated with that of BRQ primers, while others were less consistent (**Fig 6B, panels *a-e***). For example, the Cq changes in MRQ2 and MRQ3 primer pairs were highly consistent with BRQ1 and BRQ2 among all 13 samples (**Fig 6B, panel *b***), while the Cq value changes for MRQ2 and MRQ1 were less correlated (**Fig 6B, panel *a***).

**Fig 7:**
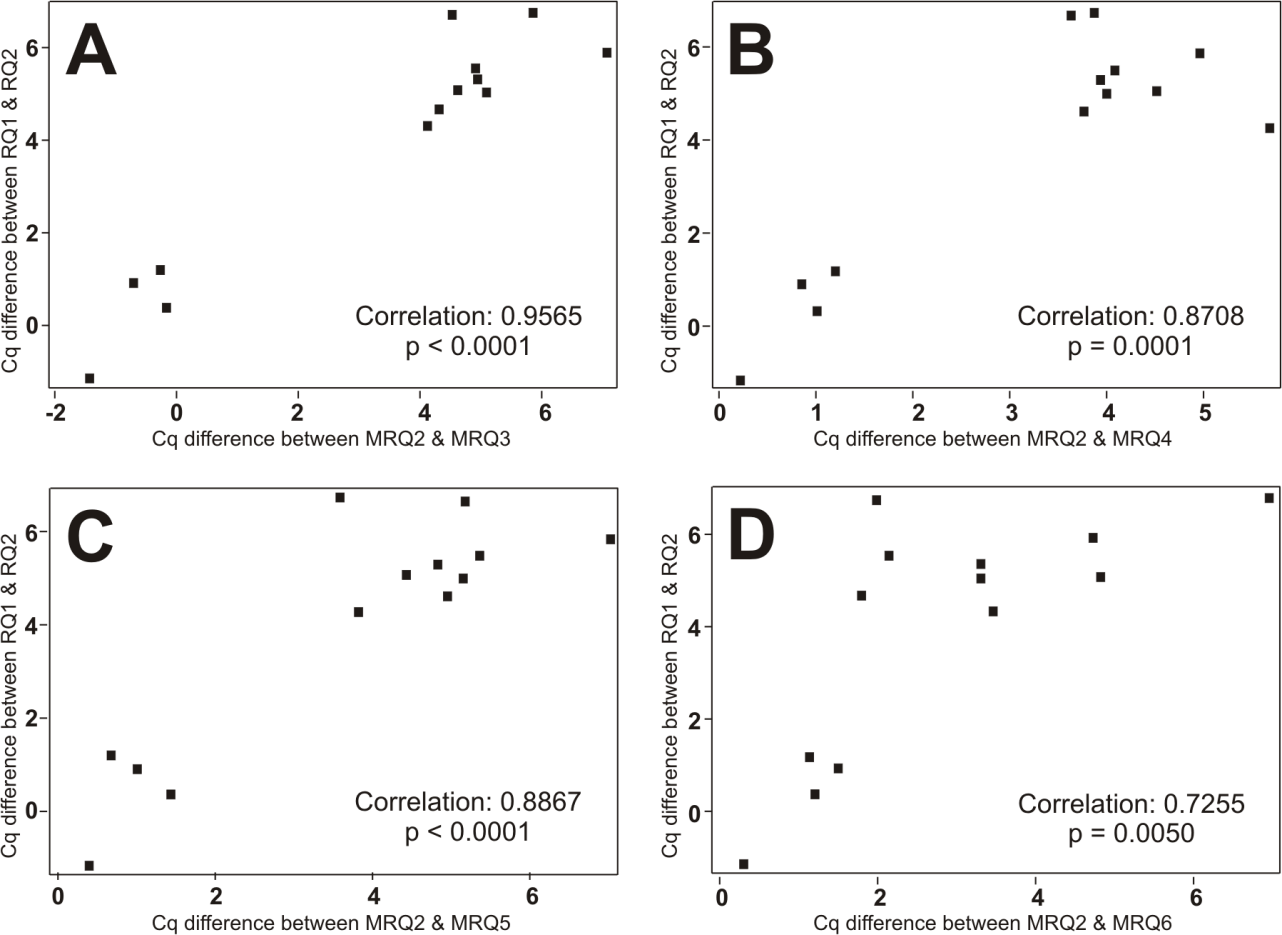
Scatter plotting of the correlation of differential Cq values between Bio-Rad’s RQ (BRQ) primers and MRQ primer pairs. Pearson correlation coefficient analysis for RQ1/RQ2 and MRQs on the 13 cDNA samples was carried out by using SAS v9.3 software. The differential Cq values between BRQ1/2 and MRQ2/3 **(A)**, MRQ2/4 **(B)**, MRQ2/5 **(C)**, MRQ2/6 **(D)**, as well as MRQ2/1 (data not shown), were all statistically positively correlated. The correlation between BRQ1/2 and MRQ2/3 is the largest, while the BRQ1/2 and MQR2/1 is the least (data not shown).

In order to identify MRQ primer pairs that may have the best correlation with the BRQ1/2 pair, we conducted the Pearson correlation coefficient analysis by comparing RQ1/RQ2 and MRQs on the 13 cDNA samples as shown in the scattering plots (**Fig 7**). We found that the different Cq values between BRQ1/2 and MRQ2/3 (**Fig 7A**, p<0.0001), MRQ2/4 (**Fig 7B**, p<0.0001), MRQ2/5 (**Fig 7C**, p<0.0001), MRQ2/6 (**Fig 7D**, p<0.005), as well as MRQ2/1 (data not shown, p = 0.0127), were all positively correlated. However, the correlation between BRQ1/2 and MRQ2/3 was the largest (R = 0.96), while the BRQ1/2 and MQR2/1 was the least (R = 0.67). Based on the Cq differences between BRQ and MRQ primer pairs, samples R1 to R4 had high quality, while the rest of the cDNA samples (e.g., R5 to R13) had much larger Cq value differences (Cq differences >4), indicating the RNA and/or cDNA quality of these samples was poor. Taken together, these results demonstrate that MRQ2/3, to a lesser extent, MRQ2/5 and MRQ2/4, can be used to effectively assess the RNA and/or cDNA quality for qPCR analysis.

## Discussion

### TqPCR effectively decreases Cq values and increases the limit of detection (LOD)

SYBR Green-based qPCR analysis of gene expression is one of the most commonly-used assays in molecular cell biology research. However, under most amplification conditions, Cq>35 usually yields less reliable results. Analytical sensitivity of qPCR usually refers to the minimum number of copies in a sample that can be measured accurately. Typically, sensitivity is expressed as the limit of detection (LOD), which is the concentration that can be detected with reasonable certainty (usually 95% probability) with a given analytical procedure [10]. While increasing the concentration of cDNA is one way to lower Cq, this can be impractical when the transcripts of interest are expressed at extremely low levels, and/or the availability of samples/cells for RNA and cDNA preparations is extremely restricted or limited.

In this report, we demonstrate that by incorporating four touchdown steps at the initial stage of qPCR analysis we are able to significantly reduce Cq values over conventional qPCR protocols. Specifically, using the sample sets of cDNA templates and/or at the same dilutions, we found that compared with the conventional qPCR protocol, our optimized TqPCR protocol can decrease the average Cq values for *Gapdh*, *Rps13*, and *Hprt1* expression by 4.45, 5.47, and 4.94 cycles, respectively. The average overall Cq value reduction for the tested three reference genes together (*Gapdh*, *Rps13*, and *Hprt1*) was 4.95. Thus, TqPCR can effectively increase the detection sensitivity, which should be highly beneficial for analyzing the expression of low-copy genes and/or cDNA samples from extremely limited quantities.

### TqPCR seemingly improves PCR amplification efficiency

Essential for relative quantification, PCR efficiency can impact the fluorescence histories and accuracy of the calculated expression results [10,61]. Individual samples may generate different fluorescence histories in kinetic qPCR. High and constant amplification efficiency in all compared samples is an important criterion for reliable comparison between samples [5,61–65]. The ideal amplification efficiency is 100%, assuming PCR product concentration doubles every cycle during the exponential phase of the reaction Practically, the amplification efficiency varies from being relatively stable in the early exponential phase and gradually declines to zero, caused by the depletion of PCR components, the decline of polymerase activity, and competition with other PCR products [5]. Thus, the PCR amplification efficiency is usually established through calibration or the use of standard curves, and the acceptable amplification efficiency range is 80% to 120% [10].

In this study, we found that TqPCR protocol improves the amplification efficiency. For example, in the mouse *Gapdh* expression analysis, the amplification efficiency was 80.9% for regular qPCR, but increased to 99.6% for TqPCR. For the *Rps13* expression analysis, the efficiency was 81.6% for qPCR, but 85.7% for TqPCR. In the case of the *Hprt1* expression, the efficiency was 114.8% for regular qPCR while the efficiency was 109.1% for TqPCR, which is closer to the acceptable norm of 100%. While we do not have a comprehensive explanation for the improved efficiency by TqPCR, it is conceivable that the initial touchdown steps may facilitate target-specific amplification and yield significant amounts of specific products, which readily serve as templates for the exponential phase of amplification. Furthermore, the above possibility may explain how TqPCR protocol effectively reduces Cq values when compared with conventional qPCR. Our findings are consistent with the previous observations, in which touchdown or step-down PCR can improve the specificity and sensitivity in PCR amplification [66,67].

### Assessment of RNA integrity and cDNA quality can be achieved by a set of homemade RQ primers

Several methods, such as *A*_260_/*A*_280_ ratio, gel electrophoresis, microfluidics-based rRNA analysis, and fluorescent RNA-binding dyes measurement, have been used to determine RNA quality, as high RNA integrity is critical for obtaining meaningful and reproducible gene expression data [2,68–72]. The reference/target gene 3’:5’ integrity assay is another useful method to determine RNA quality and cDNA integrity in one step [2]. This method involves the use two pairs of PCR primers to amplify the 3’- and 5’-end of a gene. The use of a 3’:5’ assay requires that the PCR efficiencies of both primer pairs be nearly identical [2]. Ideally, the assay should target a panel of integrity reference genes with a 3’:5’ threshold ratio of approximately 0.2–5 [10]. Bio-Rad provides pre-validated RQ1 and RQ2 primers (for mouse or human samples) to determine RNA/cDNA integrity.

In order to develop a set of RQ primers that are universally applicable and cost-effective, we characterized a panel of PCR primers derived from mouse reference gene *Tbp* and located various regions of *Tbp* mRNA. We chose the reference gene *Tbp* as the target because its expression level is considered to be at medium-low abundance, which should be sensitive enough to distinguish and validate the RNA/cDNA integrity among different samples. Using Bio-Rad’s mouse RQ primers (e.g., BRQ1 and BRQ2) as positive controls, we analyzed six primers (MRQ1-6) corresponding to mouse *Tbp* transcript on a panel of 13 cDNA samples with drastically varied RNA/cDNA integrity, and found that two overlapping primer pairs, MRQ2 and MRQ3, can distinguish the cDNA quality (Cq difference between two pairs) in the same fashion as BRQ primer pairs (R = 0.96). Other two combinations of MRQ primer pairs, MRQ2/MRQ5 and MRQ2/MRQ4, also exhibited highly significant correlations with BRQ1/BRQ2 (R = 0.89 and R = 0.87, respectively) in terms of validating the quality of the 13 tested cDNA samples. We found that the RNA integrity and cDNA quality are acceptable if the Cq differences between two MRQ primer pairs are <2. Thus, these MRQ primer pairs should be valuable resources and universally applicable for verifying RNA integrity and cDNA quality of mouse samples.

### Quantification of Wnt3A-induced target gene expression in mesenchymal stem cells validates the advantages of the TqPCR protocol

In our proof-of-principle studies, we demonstrated that the TqPCR protocol is more efficient in detecting the up-regulation of the known Wnt3A target genes in mesenchymal stem cells. Wnts are a family of secreted glycoproteins that regulate many developmental processes [73]. Wnt signaling plays an important role in skeletal development [54,74]. Wnt proteins bind to their cognate receptor frizzled (Fz) and LRP-5/6 co-receptors, and activate distinct signaling pathways, including the canonical β-catenin pathway, in which β-catenin accumulates in the cytoplasm and translocates into the nucleus where it associates with Tcf/LEF transcription factors to regulate the expression of target genes, such as Axin2 [55–57], Oct4 [58], Gbx2 [59] and NeuroD1 [60] and others [53,75–77].

We compared the Wnt3A-induced target gene expression quantified by using the conventional qPCR and TqPCR. Among these examined target genes, Axin2 has the highest abundance so both conventional qPCR and TqPCR can easily detect the up-regulation upon Wnt3A stimulation. However, for the two target genes Oct4 and Gbx2, which have relatively lower abundance, conventional qPCR failed to detect any significant induction of their expression by Wnt3A, while under the same template conditions TqPCR revealed the expression of Oct4 and Gbx2 was induced by Wnt3A for 3.1 and 7.5 times, respectively. Thus, these results further confirm that TqPCR can significantly increase the detection sensitivity of gene expression quantification. TqPCR should be especially advantageous over conventional qPCR when the transcripts of interest are lowly expressed, and/or the availability of total RNA is highly restricted or limited, such as from single cells, plasma, cell-free body fluids, laser-captured dissections, or clarified tissues.

In summary, we developed a new and effective qPCR protocol, TqPCR, by incorporating a touchdown stage prior to the quantification amplification stage. Using the same cDNA templates at the same dilutions, we found that compared with conventional qPCR, TqPCR can reduce the Cq values for *Gapdh*, *Rps13*, and *Hprt1* reference gene expression on average by 4.45, 5.47, and 4.94 cycles, respectively. The average overall Cq value reduction for the tested three reference genes was 4.95. We also found TqPCR can improve PCR efficiency and thus increase detection sensitivity. When the quantification of Wnt3A-induced target gene expression in mesenchymal stem cells was carried out, we found that TqPCR, but not conventional qPCR, was shown to effectively detect the up-regulation of lowly expressed Oct4 and Gbx2, while both conventional qPCR and TqPCR were able to detect the induced expression of the relatively abundant target Axin2. Furthermore, we evaluated and identified at least two pairs of RNA integrity/quality primers (MRQs) derived from mouse reference gene *Tbp* transcript can reliably validate RNA/cDNA integrity. Taken together, our findings demonstrate that TqPCR can increase the detection sensitivity and PCR amplification efficiency. Thus, TqPCR should be advantageous over conventional qPCR in expression quantification when the transcripts of interest are lowly expressed, and/or the availability of total RNA is highly restricted or limited.

## Supporting Information

S1 Table Primers used for PCR analysis. (XLS)

S2 Table MRQ Primers used for QR analysis. (XLS)
